# The effect of repeated preheating of dimethacrylate and silorane-based composite resins on marginal gap of class V restorations

**DOI:** 10.15171/joddd.2017.007

**Published:** 2017-03-15

**Authors:** Parnian Alizadeh Oskoee, Fatemeh Pournaghi Azar, Elmira Jafari Navimipour, Mohammad Esmaeel Ebrahimi chaharom, Fereshteh Naser Alavi, Ashkan Salari

**Affiliations:** ^1^Dental and Periodontal Research Center, Tabriz University of Medical Sciences, Tabriz, Iran; ^2^Professor, Department of Operative Dentistry, Faculty of Dentistry, Tabriz University of Medical Sciences, Tabriz, Iran; ^3^Assistant Professor, Department of Operative Dentistry, Faculty of Dentistry, Tabriz University of Medical Sciences, Tabriz, Iran; ^4^Associate Professor, Department of Operative Dentistry, Faculty of Dentistry, Tabriz University of Medical Sciences, Tabriz, Iran; ^5^Postgraduate Student, Department of Operative Dentistry, Faculty of Dentistry, Tabriz University of Medical Sciences, Tabriz, Iran; ^6^Postgraduate Student, Department of Periodontics, Faculty of Dentistry, Tabriz University of Medical Sciences, Tabriz, Iran

**Keywords:** Marginal adaptation, preheating, silorane-based composite resin

## Abstract

***Background.*** One of the problems with composite resin restorations is gap formation at resin‒tooth interface. The present study evaluated the effect of preheating cycles of silorane- and dimethacrylate-based composite resins on gap formation at the gingival margins of Class V restorations.

***Methods.*** In this in vitro study, standard Class V cavities were prepared on the buccal surfaces of 48 bovine incisors. For restorative procedure, the samples were randomly divided into 2 groups based on the type of composite resin (group 1: di-methacrylate composite [Filtek Z250]; group 2: silorane composite [Filtek P90]) and each group was randomly divided into 2 subgroups based on the composite temperature (A: room temperature; B: after 40 preheating cycles up to 55°C). Marginal gaps were measured using a stereomicroscope at ×40 and analyzed with two-way ANOVA. Inter- and intra-group comparisons were analyzed with post-hoc Tukey tests. Significance level was defined at P < 0.05.

***Results.*** The maximum and minimum gaps were detected in groups 1-A and 2-B, respectively. The effects of composite resin type, preheating and interactive effect of these variables on gap formation were significant (P<0.001). Post-hoc Tukey tests showed greater gap in dimethacrylate compared to silorane composite resins (P< 0.001). In each group, gap values were greater in composite resins at room temperature compared to composite resins after 40 preheating cycles (P<0.001).

***Conclusion.*** Gap formation at the gingival margins of Class V cavities decreased due to preheating of both composite re-sins. Preheating of silorane-based composites can result in the best marginal adaptation.

## Introduction

Improvements in the properties of modern composite resins have resulted in their use for different dental restorative procedures. The key to the clinical success of composite resin restorations is to achieve internal and marginal adaptation of the material and interfacial sealing of the cavity walls.^1^ One of the problems associated with composite resin restorations is poor adaptation and gap formation between the restorative material and the cavity walls, resulting in the microleakage of oral fluids and accumulation of fluids responsible for many problems such as postoperative hypersensitivity, marginal discoloration and recurrent caries.^2^ Factors responsible for gap formation include polymerization shrinkage stress of composite resin. The degree of shrinkage depends on the inorganic filler content of the resin, the type of the monomer system and the monomer conversion rate.^3^

Commonly used highly filled composite resins that have high viscosity pose problems in relation to placement in the cavity and adaptation.^4^ One of the techniques suggested to solve the adaptation problems and reduction of microleakage is to use a liner of flowable composite resin before placing the conventional composite resin in the cavity preparation.^5^ Flowable composite resins are not as durable as high-viscosity composite resins, due to their low filler content. In addition, the application of resin liners increases the number of procedural steps.^6^

It has recently been demonstrated that preheating of composite resins decreases their viscosity and film thickness, increasing flowability and improving their adaptation with the cavity walls.^7-11^ Choudhary et al^5^ evaluated the effect of composite resin preheating on gap formation at three different temperatures and showed better marginal seal at 54°C as compared to room temperature and 37°C. Arslan et al^12^ that reported prewarming of composite resins before polymerization could reduce microleakage values of dimethacrylate-based composite resins but could not affect the microleakage values of silorane-based composite resins.

During the preheating process, the syringe containing composite resin is prewarmed in an environment at 39‒68°C before being used.^13^ Increasing the temperature decreases viscosity, possibly affecting the polymerization kinetics and increasing the conversion rate.^14^The mobility of molecules and free radicals is influenced directly by temperature and indirectly by decreased viscosity.^15,16^ When the conversion rate of resin monomers increases, the polymerization shrinkage and consequently stresses may increase.^17,18^

In recent years, low-shrinkage resin materials that are synthesized based on siloxane and oxirane molecules’ chemistry have been introduced to overcome the problems resulting from polymerization shrinkage.^12,19^

The flowability and performance of composite resins after heat treatment is affected by the brand and the type of the preheated composite resin.^8,20^In this regard, an in vitro study showed that preheating of conventional composite resins at 54ºC and 60ºC reduces their film thickness, independent of the classification of composite resin. It has been reported that nanohybrid bulk fill composite resins exhibit the greatest reduction and microhybrid and packable exhibit the lowest reduction in film thickness.^21^In another study the film thickness of nanofill composite was not affected by preheating but the thickness of submicron hybrid composite resin showed the greatest reduction.^8^Because of variations in the chemistry and composition of composite resins, great variations are expected in viscosity after temperature increases.^6^

Despite the advantages mentioned above for composite resin preheating, the effect of thermal cycling on disintegration of polymerizing components, the mechanical properties and shelf life of composite resins should be noticed. Although many studies have shown that preheating has no detrimental effect on the mechanical properties of composite resins,^22,23^ in these studies the mechanical properties have been evaluated after only one thermal cycle; however, under clinical conditions, a syringe containing composite resin is repeatedly used for restoration of several cavities and if preheating is applied, this syringe will undergo several thermal cycles.^13^ Considering the importance of the interfacial bond between composite resin and the cavity walls, differences in the chemical structure and polymerization processes between silorane-based and dimethacrylate-based composite resins and their possible effect on the behavior of composite resin after several heat treatment procedures, the aim of the present study was to determine the effect of preheating cycles of silorane- and dimethacrylate-based composite resins on gap formation at the gingival margins of Class V cavities.

## Methods

The study protocol was approved by the Ethics Committee at Tabriz University of Medical Sciences (TBZMED.REC.1394.608)

Table 1 presents the characteristics of the materials used in the present study.

**Table 1:** The materials used in the present study

**Materials**	**Type**	**Description & Composition**
Filtek P90 (3M ESPE, St. Paul, USA)	Silorane-based microhybrid composite (Shade: A3)	Silorane resin, initiating system: Camphorquinone, Iodonium salt, Electron donor; Quartz filler, Yttrium Fluoride
Silorane adhesive system (3M ESPE, St. Paul, USA)	Two-step self-etch	Primer: Phosphorylated methacrylates, Viterbond copolymer, Bis-GMA, HEMA, Water, Ethanol, Silorane-treated silica filler; Bond: Hydrophobic dimethacrylate, Phosphorylated methacrylates, TEGDMA, Silorane-treated silica filler
Filtek Z250 (3M ESPE, St. Paul, USA)	Methacrylate-based microhybride composite (Shade: A3)	Bis-GMA, Bis-EMA, UDMA, TEGDMA, Zirconia, Silica
Clearfil SE Bond (Kuraray, Osaka, Japan)	Two-step self-etch	Primer: MDP, HEMA, water, ethanol, initiator, accelerators, dyes; Bond: MDP, HEMA, Bis-GMA, colloidal silica, initiator

Bis-EMA: bisphenol Aethoxylated dimethacrylate; Bis-GMA: bisphenol A glycol dimethacrylate; HEMA: 2-hydroxyethyl methacrylate; MDP: 10-methacryloyloxydecyl di-hydrogen phosphate; TEGDMA: triethylene glycol dimethacrylate; UDMA: urethane dimethacrylate.

The present in vitro study was carried out on 48 sound bovine incisors without any caries, cracks, fractures or anomalies in the buccogingival region. The teeth were cleaned with a rubber cup and pumice and stored in 0.5% chloramine T trihydrate solution for one week and then stored in deionized water in a refrigerator at a temperature of 4ºC.^24^ Twenty-four hours before the experimental procedures, the teeth were immersed in deionized water at a temperature of 23±2ºC.^24^ To prepare the samples, standard Class V cavities (2 mm in depth, 2 mm in mesiodistal width and 3 mm in the occlusogingival width) were prepared on the buccal surfaces with butt joint margins; the occlusal and gingival margins of the cavities were both placed 1.5 mm occlusal to and gingival to the CEJ, respectively.^25^ The cavity preparation procedures were carried out with sharp diamond fissure instruments (Diatech Dental AG Heerbrugg, Switzerland) in a high-speed handpiece with air and water cooling. Then the samples were randomly divided into 2 restorative groups based on the composite resin type (n= 24):

**Group 1:** dimethacrylate-based composite resin (Filtek Z250)

**Group 2:** silorane-based composite resin (Filtek P90)

Each group was randomly divided into 2 subgroups based on the composite resin temperature (n = 12):

**A:** composite resin at room temperature

**B:** composite resin after 40 cycles of preheating up to 55°C

In group 1-A, after irrigating and air drying the cavities, Clearfil SE Bond adhesive system was used based on manufacturer’s instructions. The self-etch primer was applied for 20 s and dried with an air syringe. The bonding agent was used, dried with an air syringe and light-cured for 10 s. Then the cavities were restored with Filtek Z250 composite resin in one layer and light-curd for 40 s with a tungsten halogen light-curing unit (Astralis 7; Ivoclar Vivadent, Liechtenstein, Austria) at a light intensity of 500 mW/cm^2^ and light-conducting tip with 8 mm light probe and perpendicular to the composite resin surface.

In group 1-B, before the application of Clearfil SE Bond adhesive system, Filtek Z250 composite resin was preheated up to 55°C in a warm thermostatically-controlled water bath (TELEDYNE HANAU, Buffalo, NY, USA). The temperature of the material was measured by a digital temperature microprobe (GBC KDM 350, KON EL CO SpA, Milano, Italy). A preliminary test was carried out to evaluate the time needed for heating and cooling the composite resin. A preheating cycle consisted of the time necessary to warm the composite resin up to 55°C and cooling it to 23°C of room temperature (each lasting 12 min in the preliminary test). The preheating cycles were repeated 40 times and the composite resin was placed in the cavity after the 40th cycle and light-cured after 15 s of delay.^26^

In group 2-A, the silorane adhesive system was applied according to manufacturer’s instructions. The self-etch primer was applied for 15 s and dried with an air syringe, followed by light-curing for 10 s. The adhesive was used for 10 s and dried with an air syringe, followed by curing for 10 s. Then the cavities were restored with Filtek P90 composite resin.

In group 2-B, before the application of silorane adhesive system, preheated Filtek P90 composite resin was used to restore the cavities in a manner similar to that in group 1-B.

Each specimen was finished and polished with the medium, fine and extra-fine disks (Sof-Lex TM, 3M ESPE Dental Products, St. Paul, USA); each disk was used 10 times for a total duration of 20s^27^directed from the composite to the tooth.^28^

All the specimens were incubated in deionized water at 37°C for 24 h. In order to simulate the oral cavity conditions, a 500-round thermocycling procedure was carried out at 5‒55°C with a dwell time of 30 s and a transfer time of 10 s in a water bath.^25^

**Figure 1.**
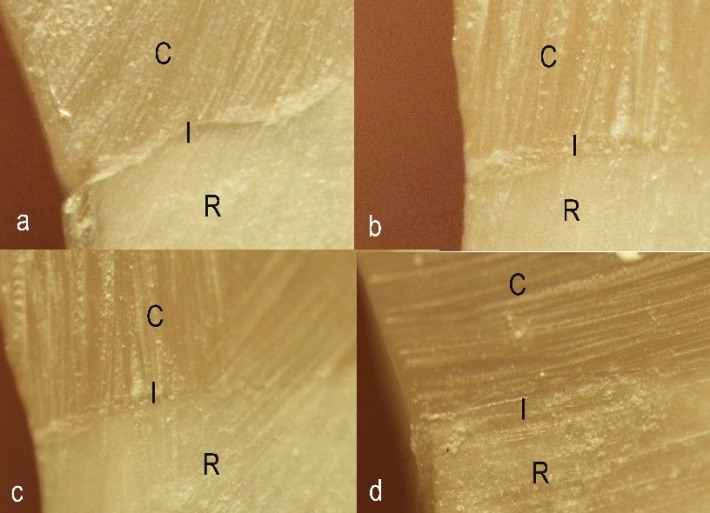
Stereomicroscope image of gap formation in group 1-A (a), group 1-B (b), group 2-A (c), and group 2-B (d).

Finally, the samples were sectioned into two halves at the middle of the restoration in a buccolingual direction, using a diamond disk (Diamant Gmbh, D&Z, Berlin, Germany). Gingival margin gaps were measured using a stereomicroscope (SMZ 1500; Nikon, Tokyo, Japan) at ×40 magnification.^24,29^A digital camera was used to photograph the selected areas with the use of a DS-L2 control unit (Nikon, Tokyo, Japan) so that the gaps could be measured.^24,29^The gap widths were measured with the built-in software in µm by determining two points on each side of the gap (one on the restoration side and one on the root side) and measuring the distance between these two points. The width of the marginal gap was measured at three points (external, middle and internal) and their means were determined as the width of the marginal gap (Figure 1).

Two-way ANOVA was applied to compare the mean gap width between the groups. Post-hoc Tukey tests were used for inter- and intra-group comparisons. Statistical significance was set at α=0.05.

## Results

Table 2 presents the means and standard deviations of marginal gap widths of silorane- and dimethacrylate-based composite resin restorations under different temperature conditions. The maximum and the minimum marginal gaps were detected in the groups restored with dimethacrylate-based composite resin at room temperature (group 1-A) and preheated silorane-based composite resin (group 2-B), respectively. Marginal gap data was analyzed using two-way ANOVA. Based on the results, both composite resin types and heat treatment had significant effects on marginal gap formation (P < 0.001); in addition, the interactive effect of these two factors on gap formation was significant (P < 0.001). Post-hoc Tukey tests showed statistically significant differences in inter- and intra-group comparisons (Table 2).

**Table 2:** The means (standard deviations) of marginal gaps in µm

**Composite resin type**	**Composite resin temperature**		**Intra-group P-value**
	**Room temperature**	**After 40 cycles of preheating**	
**Dimethacrylate**	35.9 (4.7)	20.2 (3.2)	<0.001^*^
**Silorane**	22.2 (3.3)	14.4 (2.2)	<0.001^*^
**Inter-groupP-value**	<0.001^*^	<0.001^*^	—

^*^ Statistically significant

## Discussion

The present study evaluated the effect of multiple preheating cycles before photo-polymerization of silorane- and dimethacrylate-based composite resins on gap formation at gingival margins of Class V cavities.

Based on the results, the mean gap width using both composite resin types after 40 preheating cycles was significantly less than that of using composite resins stored at room temperature. In this context, Choudhary et al^5^ and Froes-Salgado et al^30^ reported better marginal adaptation and less marginal gaps after composite resin preheating. As mentioned earlier, preheating is to warm composite resin before placing it in the cavity and photo-polymerizing it.^13^ Since composite resin is a viscoelastic material, increasing its temperature decreases its viscosity and increases its liquidity,^15^ which is attributed to thermal vibration and the subsequent separation and further sliding of monomers.^6^ In these conditions, the resin film thickness decreases and it easily adapts to the cavity walls;^31^ therefore, less gap formation can be expected after preheating.

However, increasing the composite resin temperature, followed by greater motility of radicals and monomers, can influence the conversion rate of composite resins.^32^ Stress caused by polymerization shrinkage increases as the conversion rate increases.^33^ By taking these into account, Lohbauer showed that preheating can extend detrimental effects on the margins of composite resin restorations.^34^ In other words, polymerization shrinkage, along with thermal shrinkage affects adaptation and marginal seal at preheated composite resin‒tooth interface. Elhejazi^26^ suggested a delay of 15 s before light-curing to solve such a problem. Zhao showed that a delay in light-curing preheated composite resin results in decreasing the temperature at which the conversion rate is affected, whereas the temperature is high enough to allow better wetting of the cavity walls.^31^ In clinical conditions and also in the present study, there was an interval between transferring the composite resin to the cavity, shaping and curing.

However,a study showed a decrease in the flexural strength of composite resins after 40 thermal cycles.^13^Based on the aforementioned studies and the results of other studies, repeated preheating cycles^7,35^ and prolonged duration of preheating^7^haveneither a significant effect on composite resin mechanical properties, nor a detrimental effect on the monomer component of composite resin.^7^ Therefore it is possible to explain why adaptation increases and marginal gaps decreases when both these composite resins are preheated.

Another important consideration in the results of this study is a better marginal seal of cavities restored with silorane-based composite resin compared to dimethacrylate-based composite resin, consistent with the results of studies by Bechtold et al,^36^ Nanjundasetty et al,^37^ Krifka et al^38^ and Bin Hasan and AL Saif,^39^ who reported a better performance of silorane-based composite resins in terms of marginal adaptation. However, Arslan et al^12^ did not report any differences between these two composite resin types, which was attributed to the nano-filled nature of the used dimethacrylate-based composite resin.

One of the factors affecting marginal gap formation is the chemical composition of composite resin and the polymerization mechanism. Free radical polymerization of dimethacrylate-based composite resin results in 2‒5% of volumetric shrinkage, and increased stresses can lead to debonding of the restoration material from the tooth structure at areas with a weaker bond.^40^ In silorane-based composite resins, polymerization shrinkage has been measured at 0.99% with the use of Archimedes method.^19^ In this system, photo-ring-opening-cationic polymerization technique has been used instead of free radical-mediated polymerization; this reaction begins with the ring opening systems. The process creates some spaces and consequently compensates for the contraction of chemical bonds, resulting in less stress and polymerization shrinkage.^39^

In addition, the adhesive systems used affect formation of marginal gaps in composite resin restorations.^41^ It has been reported that mild and moderate self-etch adhesives give rise to better marginal seal at dentin margins.^42^ In the present study, the self-etch adhesive used with the silorane-based composite resin has less acidity compared to Clearfil SE Bond: 2.7 and 2, respectively.^43,44^

It was concluded that in silorane-based composite resins, factors such as low viscosity and wetting ability due to preheating, inherently lower polymerization shrinkage combined with the less acidity and technique sensitivity of adhesive system,^39^result in the least marginal gap width.

It should be pointed out that it is difficult to extend the in vitro findings to the clinical action of restorative materials. In a vital tooth, the pulpal pressure and the flow of tubular fluid affect the composite resin‒tooth interface through their significant effect on the amount of moisture in dentin. Therefore, further studies are recommended that are better related to clinical conditions.

## Conclusions

Under the limitations of this study it can be concluded that:

- Gingival margin gap width in Class V cavities decreases with the use of preheated silorane- and dimethacrylate-based composite resins.
- Preheating of silorane-based composite resin before photo-polymerization can result in the best marginal adaptation.

## Acknowledgements

The authors would like to thank Dr. M. Abdolrahimi, DDS, who edited the English manuscript of this article.

## Authors’ contributions

The study was planned by PAO, FP and EJN. The literature review was performed by PAO, FNA, and AS. FNA and PAO performed the experiments and drafted the manuscript. MEEC and FNA performed the gap measurements. The statistical analyses and interpretation of data were carried out by PAO. All the authors critically revised the manuscript for intellectual content. All the authors have read and approved the final manuscript.

## Funding

The study was supported by Dental and Periodontal Research Center at Faculty of Dentistry, Tabriz University of Medical Sciences.

## Competing interests

The authors declare that they have no competing interests with regards to authorship and/or publications of this paper.

## Ethics approval

The study protocol was approved by the Ethics Committee at Tabriz University of Medical Sciences (TBZMED.REC.1394.608).
